# PTEN alleviates maladaptive repair of renal tubular epithelial cells by restoring CHMP2A-mediated phagosome closure

**DOI:** 10.1038/s41419-021-04372-6

**Published:** 2021-11-16

**Authors:** Huizhen Wang, Yifan Wang, Xin Wang, Huimi Huang, Jingfu Bao, Wenhui Zhong, Aiqing Li

**Affiliations:** 1 State Key Laboratory of Organ Failure Research, National Clinical Research Center for Kidney Disease, Division of Nephrology, Nanfang Hospital, Southern Medical University, 510515 Guangzhou, China; 2 Guangdong Provincial Key Laboratory of Renal Failure Research, Guangzhou Regenerative Medicine and Health Guangdong Laboratory, Guangzhou, China

**Keywords:** Macroautophagy, Kidney

## Abstract

Phosphatase and Tensin Homolog on chromosome Ten (PTEN) has emerged as a key protein that governs the response to kidney injury. Notably, renal adaptive repair is important for preventing acute kidney injury (AKI) to chronic kidney disease (CKD) transition. To test the role of PTEN in renal repair after acute injury, we constructed a mouse model that overexpresses PTEN in renal proximal tubular cells (RPTC) by crossing PTEN^fl-stop-fl^ mice with Ggt1-Cre mice. Mass spectrometry-based proteomics was performed after subjecting these mice to ischemia/reperfusion (I/R). We found that PTEN was downregulated in renal tubular cells in mice and cultured HK-2 cells subjected to renal maladaptive repair induced by I/R. Renal expression of PTEN negatively correlated with NGAL and fibrotic markers. RPTC-specific PTEN overexpression relieved I/R-induced maladaptive repair, as indicated by alleviative tubular cell damage, apoptosis, and subsequent renal fibrosis. Mass spectrometry analysis revealed that differentially expressed proteins in RPTC-specific PTEN overexpression mice subjected to I/R were significantly enriched in phagosome, PI3K/Akt, and HIF-1 signaling pathway and found significant upregulation of CHMP2A, an autophagy-related protein. PTEN deficiency downregulated CHMP2A and inhibited phagosome closure and autolysosome formation, which aggravated cell injury and apoptosis after I/R. PTEN overexpression had the opposite effect. Notably, the beneficial effect of PTEN overexpression on autophagy flux and cell damage was abolished when CHMP2A was silenced. Collectively, our study suggests that PTEN relieved renal maladaptive repair in terms of cell damage, apoptosis, and renal fibrosis by upregulating CHMP2A-mediated phagosome closure, suggesting that PTEN/CHMP2A may serve as a novel therapeutic target for the AKI to CKD transition.

## Introduction

Acute kidney injury (AKI) is a syndrome defined by rapid loss of renal function and occurs in ~10–15% of in-hospital patients, and >50% of patients admitted to the intensive care unit [1, 2]. AKI is mainly caused by ischemia/reperfusion (I/R) injury, sepsis, and nephrotoxic agents [1, 2]. Aside from its high mortality, AKI can trigger immediate kidney damage (referred to as acute kidney disease) or long-term damage (in the form of chronic kidney disease (CKD) and end-stage renal disease), both due to maladaptive repair [3–5]. Pathologically, AKI is mainly characterized by renal tubule epithelial cell damage and subsequent dysfunction and cell death [6, 7]. The relationship between AKI and CKD is determined by the severity of renal tubule damage; and if renal tubule damage is alleviated in the acute-injury stage and adaptive repair is facilitated, then tubular structure and function can be restored to normalcy. Notably, AKI experimental models detected altered autophagy an intracellular degradation process that is essential for physiological function and survival of renal tubular cells [8–10]. Nevertheless, whether and how autophagy flux works in the pathogenesis of renal I/R injury and repair remains controversial.

Phosphatase and Tensin Homolog on chromosome Ten (PTEN) is a critical tumor suppressor that possesses dual-specificity phosphatase. PTEN governs a variety of biological processes in both PIP3-dependent and PIP3-independent pathways, including genome maintenance, cell motility, proliferation, survival, and metabolism [11, 12]. Recent studies increasingly suggest that PTEN plays a prominent role in kidney diseases, especially diabetic kidney disease (DKD) and renal fibrosis [13, 14]. Indeed, overexpression of PTEN reportedly relieves renal fibrosis and slows CKD progression by suppressing phosphoinositide-3 kinase (PI3K)/Akt and transforming growth factor (TGF)-β-induced JNK signaling [15, 16]. In confirmation of this idea, we previously demonstrated that podocyte-specific PTEN knock-in alleviated urinary albumin and glomerular sclerosis in mice subjected to DKD [17]. Moreover, emerging evidence showed that PTEN was dysregulated in kidneys subjected to I/R injury, with abnormal PTEN expression driving cell-cycle progression and apoptosis of renal tubular cells [18, 19]. In cancer cells, PTEN reportedly regulates autophagosome biogenesis to preserve cellular integrity via PI3K/Akt/mammalian target of rapamycin signaling [20, 21]. Nevertheless, the role and mechanism of PTEN in autophagy and renal repair following acute injury remains far from clear. We hypothesized that PTEN protects the kidney against I/R-induced maladaptive repair by regulating autophagy flux.

Prior to formation of functional autolysosomes, lysosomes must be recruited and fused, which is triggered by a process where the inner and outer membranes of the autophagosome separates (phagophore closure). This process requires charged multivesicular bodies protein 2A (CHMP2A), an important component of endosomal sorting complexes for transport-III (ESCRT-III) [22, 23]. CHMP2A depletion, under both starved and non-starved conditions, causes accumulation of immature autophagosomal structures but not degradative autophagic structures (autolysosomes), reflecting an impairment of autophagy flux [22]. Nevertheless, no study to date has tested the role of CHMP2A-mediated autophagy flux in I/R-induced renal injury and repair.

To investigate the role of PTEN in I/R-induced renal maladaptive repair, we constructed mouse models where renal proximal tubular cells (RPTCs) specifically overexpressed PTEN and also cultured HK-2 cell models. Using mass spectrometry (MS)-based proteomics, we found that CHMP2A was significantly increased in RPTC-specific PTEN overexpression mice subjected to I/R. Further, we explored whether and how PTEN regulates autophagy flux via CHMP2A-mediated phagophore closure.

## Results

### PTEN was downregulated in renal tubular cells subjected to I/R-induced renal injury and maladaptive repair

**Fig. 1:**
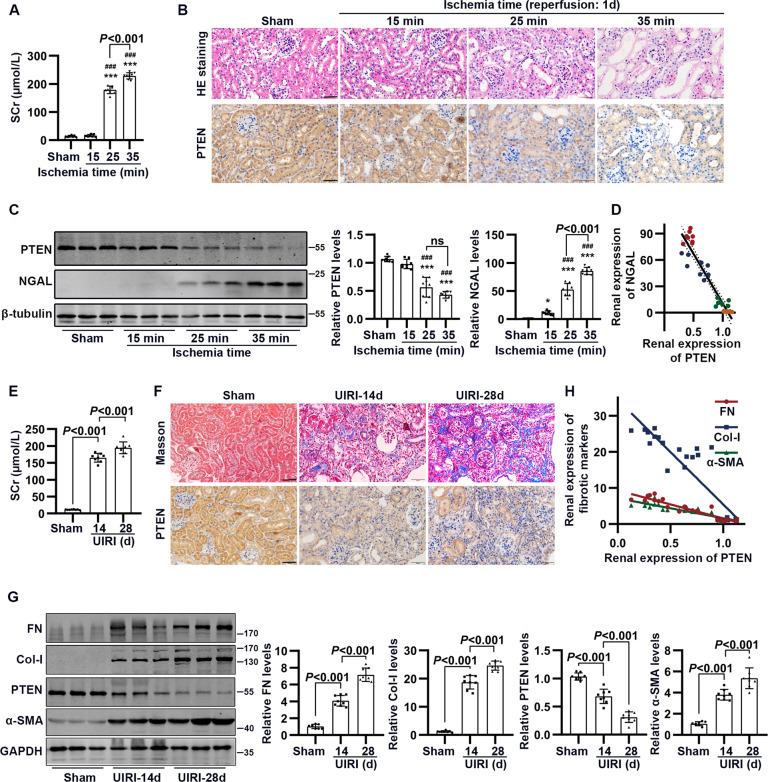
PTEN expression in kidney subjected to I/R-induced renal injury and maladaptive repair. **A** Concentration of serum creatinine (SCr) in mice subjected to bilateral renal artery obstruction at the indicated times, followed by 24 h of reperfusion (BIRI). **B** Representative HE staining and immunohistochemical staining of PTEN in kidney subjected to BIRI (scale bar, 50 μm). **C** Western blotting analysis showing changes in PTEN and NGAL in renal cortex subjected to BIRI (*P < 0.05; and ***P < 0.001 compared with values in the sham group; ^###^*P* < 0.001 compared with values in BIRI-15 min ischemia group; *n* = 8 mice in each group). **D** Correlation analysis between renal expression of PTEN and NGAL (sham, orange; BIRI-15 min, green; BIRI-25 min, blue; and BIRI-35 min, red dots). **E** Concentration of SCr in mice subjected to unilateral renal artery obstruction for 30 min, followed by 14 and 28 days of normal feeding (UIRI). **F** Representative Masson staining and immunohistochemical staining of PTEN in kidney subjected to UIRI (scale bar, 50 μm). **G** Western blotting analysis showing changes in PTEN and fibrotic proteins in renal cortex subjected to UIRI (*n* = 8 mice in each group). **H** Correlation analysis between renal expression of PTEN and fibrotic proteins.

Mice were subjected to bilateral renal artery obstruction for 15, 25, and 35 min, followed by 24 h reperfusion (BIRI). At 25 and 35 min obstruction, the level of serum creatinine (SCr) increased significantly, as compared to sham mice, consistent with the progressive renal tubular injury detected by hematoxylin and eosin (HE) staining (Fig. 1A, B and SFig. 1A). Immunoblotting and immunohistochemical analyses revealed that, at 25 and 35 min obstruction, I/R caused a significant decrease in PTEN expression (compared to sham mice) and an increased neutrophil gelatinase-associated lipocalin (NGAL) expression in the kidney, especially in tubule epithelial cells (Fig. 1B, C and SFig. 1B). Moreover, renal expression of PTEN negatively correlated with NGAL (*r* = −0.9416, 95% CI: −0.9713 to −0.8826; Fig. 1D).

In another set of experiments, mice were subjected to unilateral renal artery obstruction for 30 min, followed by 14 and 28 days of normal feeding (UIRI; SFig. 1C). In I/R-induced renal maladaptive repair mice (compared to sham mice), the level of SCr increased significantly at 14 and 28 days, consistent with progressive renal pathological changes (Fig. 1E and SFig. 1E, F). Fibrosis was obvious at 14 and 28 days post-I/R, as indicated by decreased kidney weight and increased Masson, Sirius red-positive staining, and levels of fibrotic proteins (Fig. 1F, G and SFig. 1D, E, G, H). Nevertheless, at 14 and 28 days post-I/R, PTEN protein expression in tubule epithelial cells declined progressively (Fig. 1F, G and SFig. 1I). Moreover, renal expression of PTEN negatively correlated with the levels of fibrotic proteins (fibronectin (FN): *r* = −0.9327; collagen I (Col-I): *r* = −0.8901; α-smooth muscle actin (α-SMA): *r* = −0.9060) (Fig. 1H).

### RPTC-specific PTEN overexpression alleviated renal damage and apoptosis exposed to acute injury

**Fig. 2:**
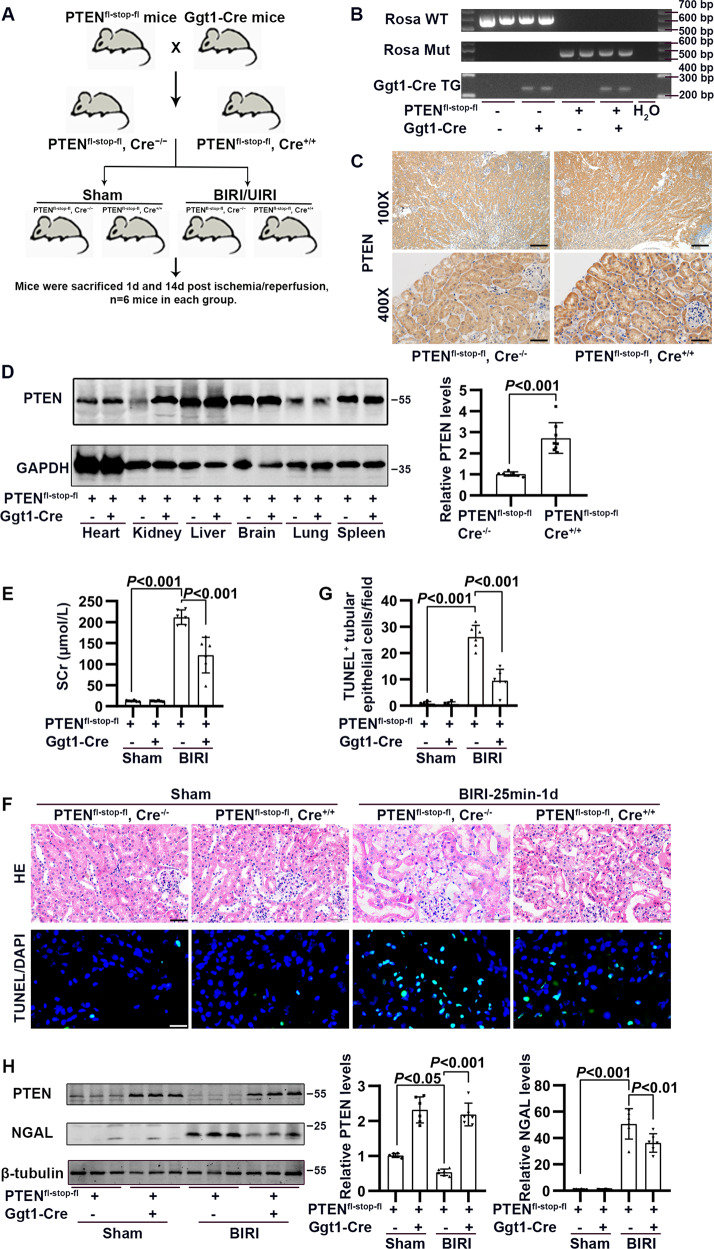
The effect of renal proximal tubular cell (RPTC)-specific PTEN overexpression on I/R-induced renal injury and apoptosis. **A** Schematic representation of renal proximal tubular cell (RPTC)-specific PTEN overexpression mice established by crossing PTEN^fl-stop-fl^ mice with Ggt1-Cre mice. **B** Identification of the genotype PTEN^fl-stop-fl^, Cre^+/+^ by Northern blotting. **C** Representative immunohistochemical staining of PTEN in kidney in PTEN^fl-stop-fl^, Cre^−/−^ and PTEN^fl-stop-fl^, Cre^+/+^ mice (scale bar: upper, 200 μm; lower, 50 μm). **D** Western blotting analysis showing changes in PTEN in different organs in PTEN^fl-stop-fl^, Cre^−/−^ and PTEN^fl-stop-fl^, Cre^+/+^ mice (*n* = 8 mice in each group). **E** Concentration of serum creatinine in PTEN^fl-stop-fl^, Cre^−/−^ and PTEN^fl-stop-fl^, Cre^+/+^ mice subjected to bilateral renal artery obstruction for 25 min, followed by 24 h of reperfusion (BIRI). **F** Representative HE staining and TUNEL staining of kidney in PTEN^fl-stop-fl^, Cre^−/−^ and PTEN^fl-stop-fl^, Cre^+/+^ mice subjected to BIRI (scale bar: upper, 50 μm; lower, 20 μm). **G** Quantification of the number of TUNEL-positive cells (*n* = 6 mice in each group). **H** Western blotting analysis showing changes in PTEN and NGAL in renal cortex in PTEN^fl-stop-fl^, Cre^−/−^ and PTEN^fl-stop-fl^, Cre^+/+^ mice subjected to BIRI (*n* = 6 mice in each group).

PTEN was overexpressed in renal tubule epithelial cells by crossing PTEN^fl-stop-fl^ mice with Ggt1-Cre mice (Fig. 2A). The genotype PTEN^fl-stop-fl^, Cre^+/+^ was confirmed by Northern blotting (Fig. 2B and SFig. 2A, B). In PTEN^fl-stop-fl^, Cre^+/+^ mice, PTEN protein expression was significantly elevated in the kidney, especially tubules, as compared to other organs (Fig. 2C, D). In tubule epithelial cells, PTEN overexpression significantly reduced SCr level and alleviated renal tubule injury, as indicated by pathologic changes and NGAL protein expression (compared with PTEN^fl-stop-fl^, Cre^−/−^ mice subjected to I/R) (Fig. 2E, F, H and SFig. 2G). In addition, in I/R-induced PTEN^fl-stop-fl^, Cre^+/+^ mice (compared to I/R-induced PTEN^fl-stop-fl^, Cre^−/−^ mice) the number of apoptotic renal tubule epithelial cells was markedly reduced (Fig. 2F, G).

### RPTC-specific PTEN overexpression facilitated tubule regeneration and alleviated renal fibrosis in maladaptive repair

**Fig. 3:**
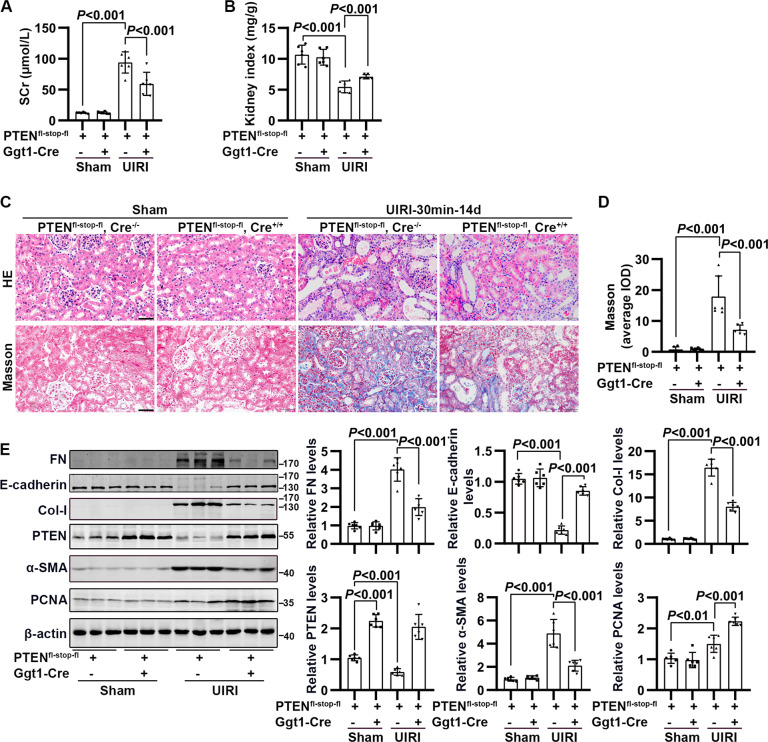
The effect of renal proximal tubular cell (RPTC)-specific PTEN overexpression on I/R-induced renal regeneration and fibrosis. **A** Concentration of serum creatinine in PTEN^fl-stop-fl^, Cre^−/−^ and PTEN^fl-stop-fl^, Cre^+/+^ mice subjected to unilateral renal artery obstruction for 30 min, followed by 14 days of reperfusion (UIRI). **B** Quantification of the ratio of kidney weight to body weight (termed the kidney index) in PTEN^fl-stop-fl^, Cre^−/−^ and PTEN^fl-stop-fl^, Cre^+/+^ mice subjected to UIRI. **C** Representative HE and Masson staining of kidney in PTEN^fl-stop-fl^, Cre^−/−^ and PTEN^fl-stop-fl^, Cre^+/+^ mice subjected to UIRI (scale bar, 50 μm). **D** Quantification of Masson-positive areas. **E** Western blotting analysis showing changes in PTEN and markers of regeneration, redifferentiation, and fibrosis in renal cortex in PTEN^fl-stop-fl^, Cre^−/−^ and PTEN^fl-stop-fl^, Cre^+/+^ mice subjected to UIRI (*n* = 6 mice in each group).

The overexpression of PTEN in tubule epithelial cells significantly reduced SCr level and the pathological damage of renal tubules and increased the kidney weight, compared to PTEN^fl-stop-fl^, Cre^−/−^ mice subjected to I/R-induced renal maladaptive repair (Fig. 3A–C and SFig. 2H). In addition, RPTC-specific PTEN overexpression increased proliferating cell nuclear antigen (PCNA) levels and E-cadherin re-expression and alleviated renal fibrosis, as indicated by reduced Masson and Sirius red-positive areas, as well as the levels of fibrotic proteins (Fig. 3C–E and SFig. 2I).

### PTEN protected from damage and apoptosis in HK-2 cells subjected to I/R treatment

**Fig. 4:**
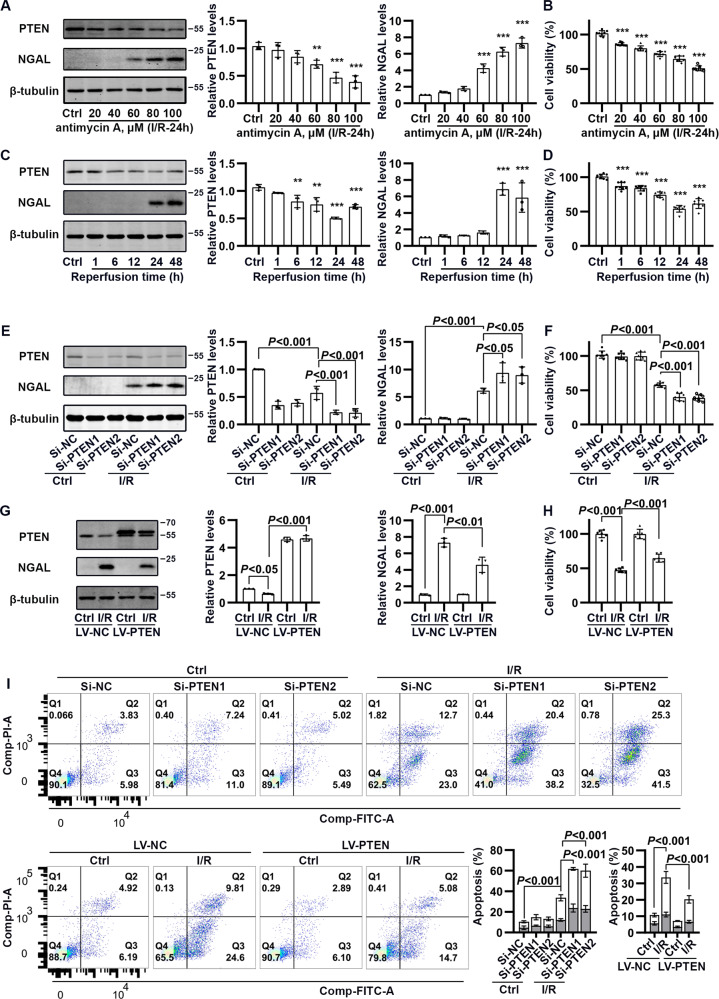
The influence of PTEN on cell damage and apoptosis of HK-2 cells in response to I/R treatment. **A** Western blotting analysis showing changes in PTEN and NGAL in I/R-treated HK-2 cells under an antimycin A concentration gradient. **B** Quantification of cell viability (%) by the CCK-8 assay in I/R-treated HK-2 cells under an antimycin A concentration gradient. **C** Western blotting analysis showing changes in PTEN and NGAL in I/R-treated HK-2 cells at the indicated reperfusion times. **D** Quantification of cell viability (%) by the CCK-8 assay in I/R-treated HK-2 cells at the indicated reperfusion times (**P < 0.01 and ***P < 0.001 compared with values in the Ctrl group). **E** Western blotting analysis showing changes in PTEN and NGAL in I/R-treated HK-2 cells in the absence and presence of si-PTEN. **F** Quantification of cell viability (%) in I/R-treated HK-2 cells in the absence and presence of si-PTEN. **G** Western blotting analysis showing changes in PTEN and NGAL in I/R-treated HK-2 cells in the absence and presence of LV-PTEN. **H** Quantification of cell viability (%) in I/R-treated HK-2 cells in the absence and presence of LV-PTEN. **I** Quantification of cell apoptosis (%) in I/R-treated HK-2 cells in the absence and presence of si-PTEN or LV-PTEN. A white rectangle represents the Q3 quadrant, and a gray rectangle represents the Q2 quadrant.

In HK-2 cells, when PTEN expression was reduced by antimycin A-mediated I/R stimulation, NGAL expression increased and cell viability decreased; and the response was dose-dependent (Fig. 4A, B). A reperfusion time-course study showed that, at 6 h reperfusion, PTEN expression levels decreased significantly; and then 24 h later, PTEN expression decreased gradually (by approximately half). Similarly, I/R caused a gradual increase in NGAL expression and decrease in cell viability, in a reperfusion time-dependent way (Fig. 4C, D). In I/R-stimulated HK-2 cells, PTEN knockdown increased NGAL expression and apoptosis and decreased cell viability. In contrast, PTEN overexpression protected HK-2 cells against I/R-induced cell damage and apoptosis (Fig. 4E–I).

### Overexpression of PTEN affected phagosome, lysosome, and response to hypoxia in I/R condition

**Fig. 5:**
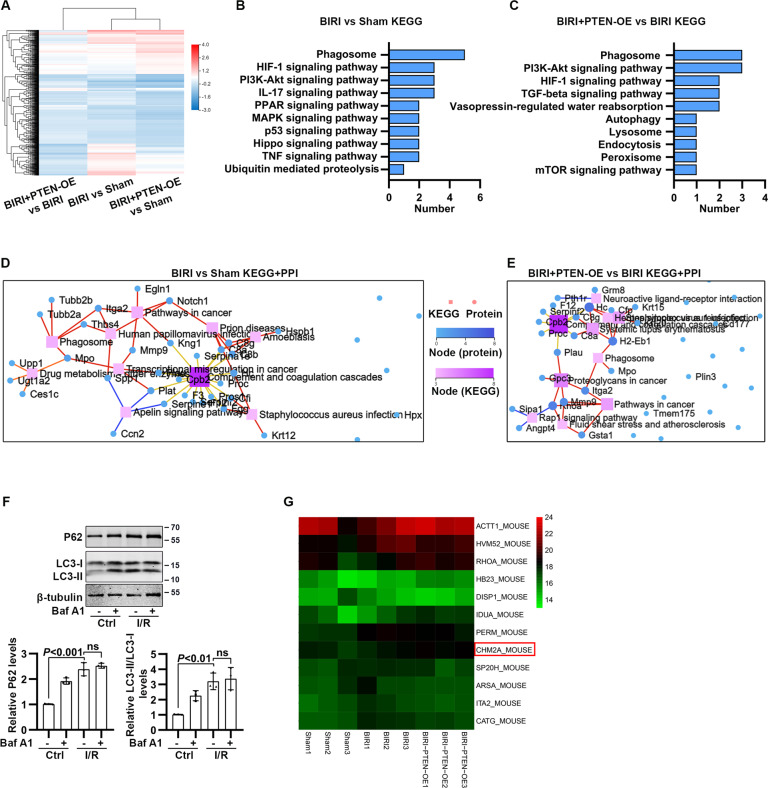
Mass spectrometry-based proteomics in I/R-induced RPTC-specific PTEN overexpression mice. **A** The heat map of renal distinct protein expression comparing sham, BIRI, and BIRI + PTEN-OE mice (*n* = 3 mice in each group). **B**, **C** The Kyoto Encyclopedia of Genes and Genomes (KEGG) pathway enrichment of differentially expressed proteins (DEPs) comparing BIRI versus sham and BIRI + PTEN-OE versus BIRI groups, respectively. **D**, **E** The KEGG pathway enrichment combined with protein–protein interaction (PPI) analysis of DEPs, comparing BIRI versus sham and BIRI + PTEN-OE versus BIRI groups, respectively. **F** Western blotting analysis showing changes in LC3 and P62 in I/R-treated HK-2 cells in the absence and presence of Baf A1. **G** The heat map of DEPs enriched in the phagosome, endocytosis, autophagy, and lysosome pathways among sham, BIRI, and BIRI + PTEN-OE mice.

A heat map based on our MS data showed distinct patterns of protein expression in the kidney when comparing sham, BIRI, and BIRI + PTEN-OE (PTEN-OE was abbreviated for PTEN^fl-stop-fl^, Cre^+/+^) mice (Fig. 5A). The Kyoto Encyclopedia of Genes and Genomes pathway combined with protein–protein interaction (PPI) analysis of differentially expressed proteins (DEPs) between BIRI and sham mice revealed that the DEPs were significantly enriched in pathways regulating the phagosome, hypoxia-inducible factor 1 (HIF-1) signaling, PI3K/Akt signaling, and interleukin-17 signaling (Fig. 5B, D). Moreover, DEPs between BIRI + PTEN-OE and BIRI mice were significantly enriched in pathways regulating the phagosome, PI3K/Akt signaling, HIF-1 signaling, and TGF-beta signaling (Fig. 5C, E). When comparing autophagy flux in I/R-treated HK-2 cells, we found that I/R caused a significant increase in the levels of LC3-II/LC3-I, accompanied with P62 accumulation. Nevertheless, bafilomycin A1 (Baf A1), an inhibitor of autophagy flux, failed to further increase LC3-II/LC3-I and P62 expression in I/R-treated HK-2 cells, indicating that autophagy flux was blocked in response to I/R (Fig. 5F and SFig. 3A). Interestingly, the heat maps of DEPs enriched in the phagosome, endocytosis, autophagy, and lysosome pathways indicated that CHMP2A, an autophagy flux-related protein, was significantly increased in BIRI + PTEN-OE mice compared to BIRI mice (Fig. 5G).

### PTEN deficiency blocked phagosome closure via downregulating CHMP2A in HK-2 cells subjected to I/R treatment

**Fig. 6:**
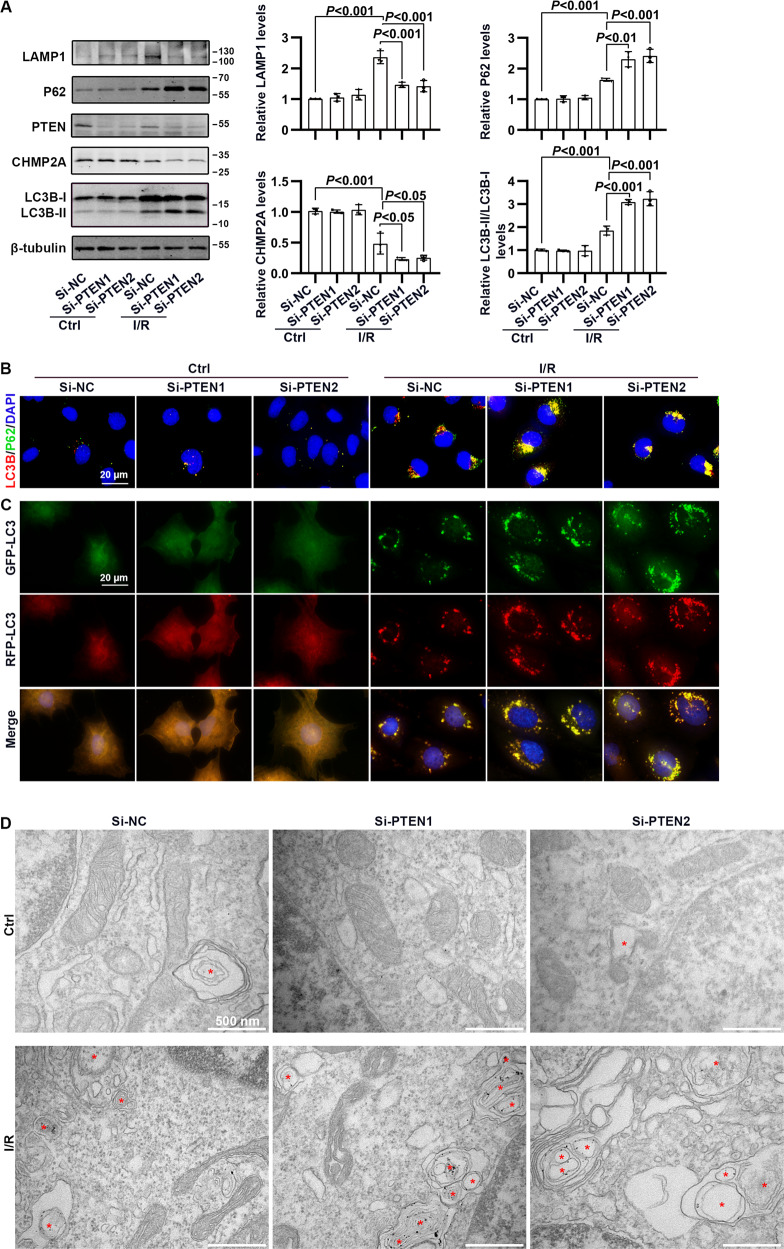
The influence of PTEN deficiency on CHMP2A-mediated autophagy flux in HK-2 cells subjected to I/R. **A** Western blotting analysis showing changes in CHMP2A and autophagy flux-related proteins in I/R-treated HK-2 cells in the absence and presence of si-PTEN. **B** Representative dual fluorescence of LC3 and P62 staining in I/R-treated HK-2 cells in the absence and presence of si-PTEN (scale bar, 20 μm). **C** Representative immunofluorescence images of RFP-GFP-LC3 in I/R-treated HK-2 cells in the absence and presence of si-PTEN, which allowed differentiation between autophagosomes (RFP^+^GFP^+^, yellow puncta) and autolysosomes (RFP^+^GFP^−^, red puncta; scale bar, 20 μm). **D** Representative transmission electron microscopic images of autophagic structures in I/R-treated HK-2 cells in the absence and presence of si-PTEN (scale bar, 500 nm).

Immunoblotting and immunofluorescence of HK-2 cells subjected to I/R revealed that PTEN knockdown significantly decreased the expression of CHMP2A and lysosome-associated membrane glycoprotein 1 (LAMP1) and accumulated autophagosome-bound LC3-II and P62 (Fig. 6A, B). Further, RFP-GFP-LC3 was applied to assess autophagy flux, to distinguish non-degradative (RFP^+^GFP^+^: yellow dots, reflecting autophagosomes) vs. degradative autophagic structures (RFP^+^GFP^−^: red dots, reflecting autolysosomes); and PTEN knockdown dramatically increased the number of RFP^+^GFP^+^ dots but not RFP^+^GFP^−^ dots in I/R-treated HK-2 cells (Fig. 6C). Simultaneously, transmission electron microscope (TEM) revealed severe structural damage and an increased number of autophagosome in I/R-treated HK-2 cells in the presence of si-PTEN (Fig. 6D).

### PTEN overexpression promoted phagosome closure by upregulating CHMP2A in HK-2 cells and mice subjected to I/R

**Fig. 7:**
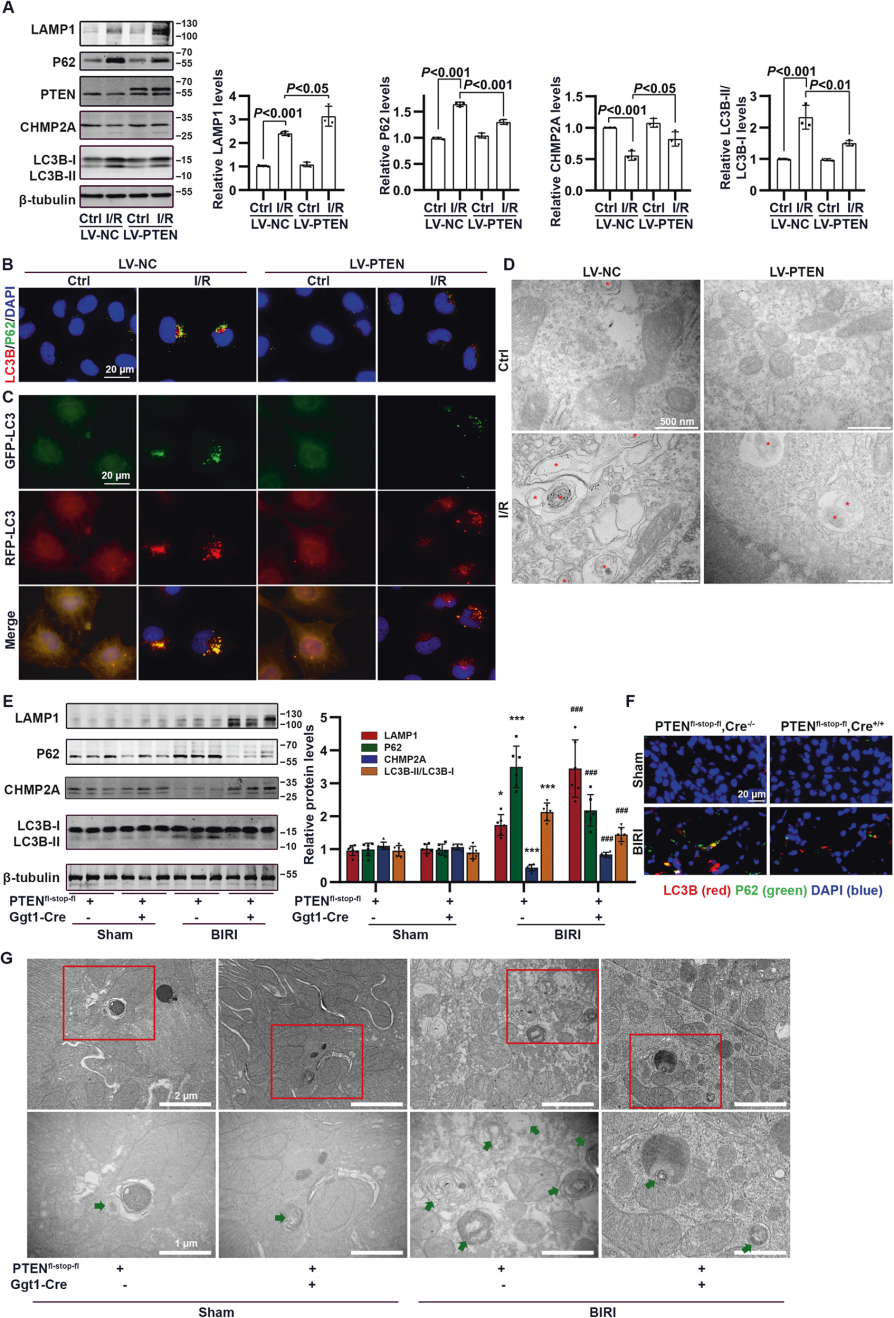
The influence of PTEN overexpression on CHMP2A-mediated autophagy flux in HK-2 cells and mice subjected to I/R. **A** Western blotting analysis showing changes in CHMP2A and autophagy flux-related proteins in I/R-treated HK-2 cells in the absence and presence of LV-PTEN. **B** Representative dual fluorescence of LC3 and P62 staining in I/R-treated HK-2 cells in the absence and presence of LV-PTEN (scale bar, 20 μm). **C** Representative immunofluorescence images of RFP-GFP-LC3 in I/R-treated HK-2 cells in the absence and presence of LV-PTEN. RFP^+^GFP^+^, yellow puncta represent autophagosomes; and RFP^+^GFP^−^, red puncta represent autolysosomes (scale bar, 20 μm). **D** Representative transmission electron microscopic (TEM) images of autophagic structures in I/R-treated HK-2 cells in the absence and presence of LV-PTEN (scale bar, 500 nm). **E** Western blotting analysis showing changes in CHMP2A and autophagy flux-related proteins in PTEN^fl-stop-fl^, Cre^−/−^ and PTEN^fl-stop-fl^, Cre^+/+^ mice subjected to BIRI (*P < 0.05 and ***P < 0.001 compared with values in sham + PTEN^fl-stop-fl^, Cre^−/−^ group; ^###^*P* < 0.001 compared with values in BIRI + PTEN^fl-stop-fl^, Cre^−/−^ group; *n* = 6 mice in each group). **F** Representative dual fluorescence of LC3 and P62 staining in PTEN^fl-stop-fl^, Cre^−/−^ and PTEN^fl-stop-fl^, Cre^+/+^ mice subjected to BIRI (scale bar, 20 μm). **G** Representative TEM images of autophagic structures (green arrows) in PTEN^fl-stop-fl^, Cre^−/−^ and PTEN^fl-stop-fl^, Cre^+/+^ mice subjected to BIRI (scale bar: upper, 2 μm; lower, 1 μm).

In HK-2 cells subjected to I/R treatment, PTEN overexpression significantly increased CHMP2A and LAMP1 expression levels and attenuated the accumulation of autophagosome-bound LC3-II and P62 but accelerated autolysosome formation (as assessed by RFP-GFP-LC3 and TEM; Fig. 7A–D).

Consistently, in I/R mice, RPTC-specific PTEN overexpression caused a dramatic increase in CHMP2A and LAMP1 expression and a decrease in autophagosome-bound LC3-II and P62 (Fig. 7E, F). In addition, the number of autophagosomes was reduced, and autolysosome was observed in PTEN^fl-stop-fl^, Cre^+/+^ mice (compared with PTEN^fl-stop-fl^, Cre^−/−^ mice exposed to I/R; Fig. 7G).

### Silencing CHMP2A abolished the effect of PTEN on phagosome closure, independent of Akt signaling

**Fig. 8:**
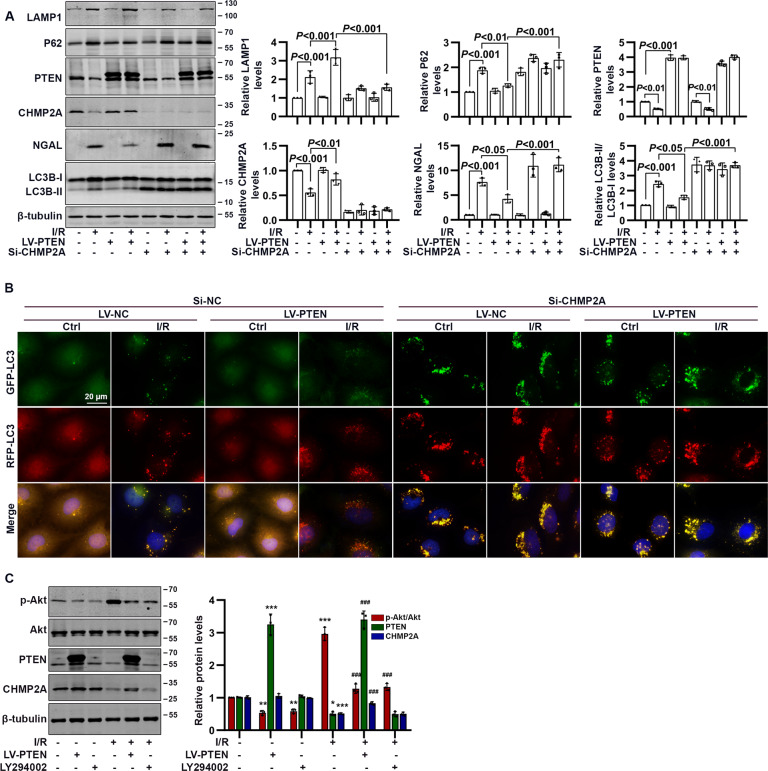
The influence of PTEN overexpression on phagosome closure in I/R-treated HK-2 cells in the absence and presence of si-CHMP2A. **A** Western blotting analysis showing changes in autophagy flux-related proteins in CHMP2A-deficient HK-2 cells exposed to I/R in the absence and presence of LV-PTEN. **B** Representative immunofluorescence images of RFP-GFP-LC3 in CHMP2A-deficient HK-2 cells exposed to I/R in the absence and presence of LV-PTEN. RFP^+^GFP^+^, yellow puncta represent autophagosomes; and RFP^+^GFP^−^, red puncta represent autolysosomes (scale bar, 20 μm). **C** Western blotting analysis showing changes in CHMP2A and Akt pathway in I/R-treated HK-2 cells exposed to LV-PTEN or LY294002 (*P < 0.05, **P < 0.01, and ***P  < 0.001 compared with values in the Ctrl group; ^###^*P* < 0.001 compared with values in the I/R group).

In normal, cultured HK-2 cells, silencing CHMP2A increased the abundance of autophagosome-bound LC3-II and P62, without increasing the number of autolysosomes (Fig. 8A, B). In I/R-treated HK-2 cells, silencing CHMP2A aggravated the accumulation of autophagosome-bound LC3-II and P62 and elevated NGAL expression. Importantly, in the presence of si-CHMP2A, overexpression of PTEN failed to promote autophagy flux and reduce NGAL expression (Fig. 8A). Consistently, fluorescent RFP-GFP-LC3 showed that silencing CHMP2A significantly increased the number of autophagosomes (RFP^+^GFP^+^ dots) in the absence and presence of LV-PTEN, under control and I/R conditions (Fig. 8B). Additionally, CHMP2A was upregulated in I/R-treated HK-2 cells exposed to LV-PTEN rather than LY294002, an inhibitor of PI3K (Fig. 8C).

## Discussion

Previous reports showed HIF-1α expression increases in response to I/R and that elevated levels of HIF-1α induce miR-21, miR-687, and other miRNAs that target PTEN in hypoxic/ischemic-treated tubular epithelial cells, inducing PTEN downregulation [18, 19, 24]. Other reports showed that, in aristolochic acid-induced kidney injury, activated nuclear factor-κB induces upregulation of miR-382, which targets the pten 3′-untranslated region, to downregulate PTEN protein levels [16]. In our study, after 15 min of kidney ischemia with renal adaptive repair, PTEN expression did not fall significantly; however, after 25 to 35 min of ischemia, PTEN did decrease significantly and renal repair became maladaptive. Consistent with these observations, in I/R-treated HK-2 cells exposed to antimycin A, PTEN levels were reduced and in a concentration-dependent manner. In addition, renal expression of PTEN negatively correlated with NGAL and fibrotic markers. This evidence suggests that PTEN protein levels, and their decrease in response to prolonged ischemia, were associated with the severity of renal injury and maladaptive repair.

Further, we demonstrated that RPTC-specific PTEN overexpression could alleviate acute renal injury and facilitate renal adaptive repair. Consistent with these data, PTEN knockdown aggravated injury and apoptosis in I/R-treated HK-2 cells, whereas PTEN overexpression restored I/R injury. In contrast, PTEN was proposed to be upregulated in kidney in I/R mice; and suppressing PTEN expression stimulated Akt phosphorylation and inhibited caspase-3 activation, triggering an anti-apoptotic effect [25, 26]. Here we propose that use of different mouse strains and the severity of injury might primarily explain the apparent contradiction in these data. During renal repair, there are two populations of tubules: one is impaired, with fibrotic tubules that fail to regenerate and redifferentiate, expressing little or no PTEN and showing expression of fibrotic genes; the other is composed of healthy tubules, plus impaired tubules, which regenerate and redifferentiate normally, and express increased levels of PTEN that extends beyond the foci of tubulointerstitial fibrosis [14, 15, 27]. Taken together, it appears that stable PTEN expression is essential for tubule epithelial cell regeneration and redifferentiation and that PTEN plays a renoprotective role in the early stage of AKI and during the progression of AKI to CKD.

To examine how PTEN protects against renal injury and maladaptive repair, we conducted MS-based proteomics comparing sham, BIRI, and BIRI + PTEN-OE mice and found that the autophagy pathway showed significant enrichment. In I/R-treated HK-2 cells, Baf A1 failed to further increase LC3-II/LC3-I and P62 levels. Notably, an increase in LC3B-II/LC3B-I can be attributed to either inhibition of autophagy flux or promotion of autophagosome biogenesis [28]. P62/SQSTM1, an autophagosome adapter, is continually degraded by the autolysosome but accumulates when autophagy flux is inhibited [29]. It was well acknowledged that, in I/R-AKI, autophagy is activated for scavenging and recycling of damaged macromolecules, which protects tubules from degeneration [30]. Nevertheless, accumulation of autophagosome-bound LC3-II and P62, and ubiquitin-positive cytoplasmic inclusions, increases apoptosis and oxidative stress and consequently exacerbates progression of AKI to CKD [31]. The above evidence suggests that, as a means to protect tubules from I/R injury and maladaptive repair, one strategy might be to enhance autophagy flux by targeting the autophagosome–autolysosome pathway, rather than stimulating autophagosome formation alone. Our study in HK-2 cells and mice showed that I/R caused autophagosome-bound LC3-II and P62 to accumulate, while autolysosomes did not accumulate. Overexpression of PTEN decreased the level of autophagosome-bound LC3-II and P62 and increased the level of autolysosomes, preventing P62 accumulation and attenuating I/R-induced cell damage; however, how PTEN is involved in autophagy flux in response to I/R remains unknown.

Using MS analysis, we profiled the autophagy-related proteins and discovered that CHMP2A was downregulated in BIRI, whereas RPTC-specific PTEN overexpression caused a significant increase in CHMP2A expression. CHMP2A is a member of the SNF7 family and is involved in protein sorting and transport from endosome to vacuole/lysosome [22]. CHMP2A is essential for annular fusion, including nuclear envelope formation of cell division, viral membrane abscission from infected cells, and autophagosome closure [22, 32, 33]. During autophagy, CHMP2A translocates to the phagophore and separates the inner and outer autophagosomal membranes to form double-membrane autophagosomes, a process required for autolysosome formation [22]. Notably, any unclosed autophagosomal membranes serve as a platform for intracellular death-inducing signaling complexes (iDISCs) [34]. Thus, silencing CHMP2A induces unclosed autophagosome-bound LC3-II and p62 accumulation and prevents lysosome recruitment and fusion by inhibiting phagophore closure, resulting in iDISC-mediated cell death [35]. These data suggest that a therapeutic approach might target autophagy at the step where autophagosomes become sealed and autolysosome form. Thus, CHMP2A may represent an attractive therapeutic target for maintaining cell integrity through promoting cytoprotective lysosomal degradation and alleviating iDISC-mediated apoptosis. Interestingly, the Genotype-Tissue Expression database (GTEx) showed a correlation between PTEN and CHMP2A in the kidney cortex (Pearson *R* = 0.6, *P* = 0.00065; SFig. 3B). In our study in HK-2 cells and mice subjected to I/R, PTEN overexpression boosted CHMP2A protein levels and this restored phagophore closure, resulting in reduced NGAL expression and apoptosis. Moreover, in I/R-treated HK-2 cells, silencing CHMP2A abolished the effect of PTEN on phagophore closure and cell damage.

How, then, does PTEN regulate CHMP2A expression? A key experiment using an inhibitor of PI3K, LY294002, showed that PTEN regulated CHMP2A independent of PI3K/Akt signaling. In addition, co-immunoprecipitation experiments found no evidence of any PPI between PTEN and CHMP2A (SFig. 3C). Interestingly, however, after I/R was induced in HK-2 cells, we found that nuclear PTEN expression was more significantly downregulated than cytoplasmic PTEN expression, suggesting that nuclear PTEN might play a pivotal role (data not shown). The translocation of PTEN to the nucleus is strongly associated with the function and activity of PTEN; and PTEN translocates to the nucleus in response to oxidative stress via activated ATM (ataxia telangiectasia mutated serine/threonine kinase)-mediated phosphorylation at serine 113 [12, 20, 36]. Once in the nucleus, PTEN activates the p-JUN-SESN2/AMPK pathway and thus induces autophagy [20, 36]. Our data together with previous reports suggest that cytoplasmic PTEN might be modified and then translocate to the nucleus where, under normal conditions, it regulates the transcriptional activity of CHMP2A. Moreover, low levels of cytoplasmic PTEN might, in turn, generate less nuclear translocation and finally reduce CHMP2A expression in I/R-exposed tissue. However, how PTEN precisely regulates CHMP2A requires further investigation.

**Fig. 9:**
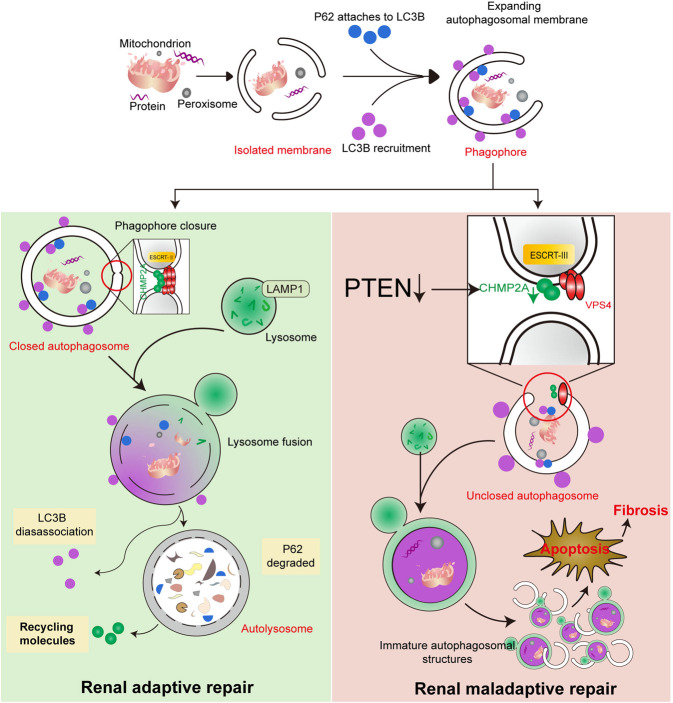
Schematic representation of PTEN regulated CHMP2A-mediated phagosome closure in I/R-induced renal maladaptive repair. During autophagy, endosomal sorting complexes required for the transport (ESCRT)-III component, CHMP2A, translocates to the expanding autophagosomal membrane and regulates the separation of the inner and outer autophagosomal membranes in the formation of double-membrane autophagosomes. CHMP2A-mediated phagophore closure is critical for functional autolysosome formation, by preventing mis-localization of lysosome-associated membrane glycoprotein 1 (LAMP1) to the inner autophagosomal membrane. Nevertheless, downregulation of PTEN reduced CHMP2A protein levels and thus impaired the ESCRT-mediated membrane abscission. This resulted in accumulation of immature autophagosomal structures, which led to cell injury, apoptosis, and fibrosis, as indicated by ischemia/reperfusion (I/R)-induced renal maladaptive repair.

Our data suggest that, during autophagy, CHMP2A translocates to the expanding autophagosomal membrane and regulates the separation of the inner and outer autophagosomal membranes and the formation of double-membrane autophagosomes. For functional autolysosomes to form, CHMP2A-mediated phagophore closure is critical because it prevents LAMP1 from being mislocalized to the inner autophagosomal membrane. Nevertheless, our work shows that, in response to I/R, PTEN was downregulated in RPTCs and downregulation of PTEN reduced CHMP2A protein levels, thus impairing ESCRT-mediated membrane abscission, resulting in accumulation of immature autophagosomal structures, cell injury, apoptosis, and fibrosis (Fig. 9).

Overall, our studies show that, if kidneys exposed to I/R-induced renal injury respond with maladaptive repair, then PTEN is downregulated; and this effect could be linked to the severity of I/R. However, RPTC-specific PTEN overexpression alleviated I/R-induced renal injury and improved renal maladaptive repair. Importantly, PTEN overexpression could promote autophagy flux by upregulating CHMP2A-mediated phagosome closure and thus attenuate cell injury and apoptosis in vivo and in vitro. This provides new insights into innovative, clinical applications that might target autophagy flux as a therapy for renal maladaptive repair.

## Materials and methods

### Transgenic mice

Transgenic B6.Cg-Tg(Ggt1-cre)M3Egn/J mice that expressed Cre recombinase under the control of *gamma-glutamyltransferase 1* (*Ggt1*) promoter uniquely in cortical proximal tubules were purchased from The Jackson Laboratory [37]. Transgenic PTEN^fl-stop-fl^ mice were established as previously described [17]. Briefly, a transcriptional stop cassette, flanked by loxP recombination sites, was constructed before the recombinant PTEN coding region and inserted into the Rosa 26 locus, which allows overexpressing of PTEN upon introduction of the Cre recombinase. RPTC-specific PTEN overexpression mice were produced by crossing PTEN^fl-stop-fl^ mice with Ggt1-Cre mice, such that Cre-mediated recombination resulted in deletion of the transcriptional stop cassette and subsequent overexpression of PTEN in cortical tubular epithelium (genotype PTEN^fl-stop-fl^, Cre^+/+^). PTEN^fl-stop-fl^ mice served as control mice (genotype PTEN^fl-stop-fl^, Cre^−/−^). All animals were born at the expected Mendelian frequency, were normal in size, and did not display any gross physical or behavioral abnormalities (SFig. 2C–F). All animal experiments were approved by the Institutional Animal Care and Use Committee of Nanfang Hospital.

### Mouse models of AKI and repair

AKI and repair was induced by renal I/R in male mice weighing ~22 g [38, 39]. In one set of mice, bilateral renal pedicles were clamped at the indicated times (15, 25, 35 min; *n* = 8 mice in each group) using microaneurysm clamps. After removal of clamps, kidney reperfusion was visually confirmed. During surgery, body temperature was maintained between 36 and 37 °C, using a temperature-controlled heating cushion (CINONTECH, China). Blood and tissue samples were obtained at 24 h reperfusion. In another set of mice, unilateral renal artery obstruction combined with contralateral uninephrectomy was utilized, and the resulting AKI repair was monitored. Briefly, left renal pedicle was clamped for 30 min and followed by reperfusion. Right kidney was excised at 13 or 27 days post-I/R. Blood and left kidney tissue were obtained at 14 or 28 days post-I/R (*n* = 8 mice in each group). In addition, PTEN^fl-stop-fl^, Cre^−/−^ and PTEN^fl-stop-fl^, Cre^+/+^ mice were randomly assigned to the sham and BIRI/UIRI groups (*n* = 6 mice in each group).

### HK-2 cell culture and treatment

A normal human proximal tubule epithelial cell line (HK-2, ATCC Cat# CRL-2190, RRID:CVCL_0302) was cultured in Dulbecco’s Modified Eagle Medium/F12 (Gibco) medium supplemented with 10% fetal bovine serum (Corning, USA) and 1% penicillin/streptomycin stock solution (10,000 U/ml). The I/R-induced HK-2 cell injury model was established by ATP/Glucose depletion [40]. In brief, when HK-2 cells were approximately 80% confluent, the culture medium was replaced with Hank’s Balanced Salt Solution (without Ca^2+^, Mg^2+^, 88284, Gibco) and then incubated with 80 µM antimycin A (Sigma) plus 10 mM 2-deoxy-d-glucose (HY-13966, MedChemExpress, MCE) for 1 h, to induce ischemic injury. In vitro reperfusion was achieved by incubating cells in complete growth medium.

Small interfering RNA (PTEN, CHMP2A) (RiboBio, China) was transfected at a final concentration of 50 nM using the Lipofectamine 3000 reagent (Invitrogen), according to the manufacturer’s instruction. PTEN was overexpressed via transfecting with a lentivirus incorporating PTEN recombinant plasmid (pHBLV-h-PTEN-3xflag-PURO, HANBIO, China), hereafter referred to as LV-PTEN. LV-NC, a lentivirus incorporating an empty plasmid, served as negative control. To generate RFP-GFP-LC3 HK-2 cells, HK-2 cells were transduced with lentiviruses encoding hU6-MCS-Ubiquitin-stubRFP-senseGFP-LC3-IRES-puromycin and selected with 1 µg/ml puromycin for 5 days. Baf A1 (100 nM, HY-100558, MCE) or LY294002 (10 μM, HY-10108, MCE) was added 2 h prior to I/R intervention and maintained until the end of the experiments.

### Renal function analysis

SCr concentration was measured by an automated chemistry analyzer (AU480; Beckman Coulter, Brea, CA, USA).

### Histology and immunohistochemical staining

Kidney samples were fixed in 10% neutral buffered formalin, paraffin-embedded, and sectioned at ~2–4 μm thickness prior to dewaxing and HE or Masson’s trichrome staining, according to the manufacturer’s protocols, respectively. Moreover, additional sections were used for immunohistochemical detection of PTEN. The percentage areas of fibrosis and positive protein immunostaining were quantified and calculated as the ratio of Masson and protein immunostaining-positive areas relative to the whole field (×400; % area), using Image-Pro Plus. This was performed by two individuals, blinded to group allocation. At least ten randomly chosen fields were evaluated for each mouse.

### Analysis of apoptosis

Apoptosis was measured by terminal deoxynucleotidyl transferase (TdT) dUTP nick-end labeling (TUNEL, Roche, USA) staining and Annexin V and propidium iodide-labeling flow cytometry (BD Biosciences, 556547), according to the manufacturers’ instructions. The apoptosis of tubule epithelial cells was assessed by determining the ratio of the number of TUNEL-positive tubular cells to the number of total cells. Flow cytometry was tested by BD LSR Fortessa, and data were analyzed by the FlowJO software. In addition, cell viability was determined by the Cell Counting Kit-8 Assay (DOJINDO, Japan) according to the manufacturer’s protocol.

### Mass spectrometry

Using MS, the proteomic profile of renal tissue was compared in sham, BIRI mice (subjected to bilateral renal artery obstruction for 25 min, followed by 24 h reperfusion), and BIRI + PTEN-OE mice (PTEN^fl-stop-fl^, Cre^+/+^ subjected to BIRI), following these steps: protein sample preparation, trypsin digestion, IBT labeling, high-performance liquid chromatography (HPLC) separation, and LC with tandem MS.

### Western blotting analysis

Western blotting was performed as previously described [17]. The primary antibodies used were as follows: anti-PTEN (Abcam Cat# ab32199, RRID:AB_777535; Cell Signaling Technology Cat# 9188, RRID:AB_2253290; Proteintech Cat# 60300-1-Ig, RRID:AB_2881415), anti-NGAL (Abcam Cat# ab63929, RRID:AB_1140965), anti-FN (Sigma-Aldrich Cat# F3648, RRID:AB_476976), anti-Col-I (Boster Biological Technology Cat# BA0325, RRID:AB_2891224), anti-α-SMA (Sigma-Aldrich Cat# A5228, RRID:AB_262054), anti-PCNA (Proteintech Cat# Biotin-60097, RRID:AB_2883063), anti-E-Cadherin (Cell Signaling Technology Cat# 14472, RRID:AB_2728770), anti-Flag (Proteintech Cat# 20543-1-AP, RRID:AB_11232216), anti-CHMP2A (Proteintech Cat# 10477-1-AP, RRID:AB_2079470), anti-SQSTM1/p62 (Cell Signaling Technology Cat# 39749, RRID:AB_2799160), anti-LC3B (Cell Signaling Technology Cat# 83506, RRID:AB_2800018), anti-LAMP1 (Cell Signaling Technology Cat# 15665, RRID:AB_2798750), anti-β-tubulin (Tianjin Sungene Biotech Cat# KM9003, RRID:AB_2744678), and anti-GAPDH (Tianjin Sungene Biotech Cat# KM9002, RRID:AB_2721026). Western blotting was performed at least three times, independently. Quantification was performed by measuring the signal intensity using the software ImageJ (National Institutes of Health).

### Immunofluorescence

For RFP-GFP-LC3 fluorescence, HK-2 cells transferred with RFP-GFP-LC3 virus on coverslips were washed 3 times with phosphate-buffered saline, fixed with 4% paraformaldehyde for 10 min, and mounted with 4,6-diamidino-2-phenylindole (DAPI). In addition, for LC3 and P62 two-color immunofluorescent staining, HK-2 cells on coverslips were fixed, permeabilized, and co-incubated with anti-LC3B and anti-P62 antibody at 4 °C overnight, followed by the corresponding Alexa Fluor secondary antibodies, and mounted with DAPI. Fluorescent images were obtained using an OLYMPUS IX81 deconvolution microscope (×63 oil-immersion lens).

### Transmission electron microscope

Briefly, renal tissue or HK-2 cells were fixed in 2.5% glutaraldehyde, washed in cacodylate buffer, and postfixed in 1% osmium tetroxide. Specimens were then dehydrated, critical-point dried in carbon dioxide, and coated with atomic gold particles. Autophagic structures were observed using a TEM (Hitachi H-7500).

### Statistical analysis

All data are presented as the mean ± SD. One-way analysis of variance followed by the Bonferroni multiple-comparison test was performed to compare parametric values among three or more groups. Correlation analysis between renal expression of PTEN and other parameters was performed using the Pearson Product-Moment Correlation. Statistical analysis of data was performed using Graph-Pad Prism 8.0 (Graph Pad Software, San Diego, CA). A *P* value <0.05 was considered statistically significant.

## Supplementary information

Supplementary material

## Data Availability

All data generated or analyzed during this study are included in this published article and its Supplementary Information files.
