# Supplementary material for: PTEN alleviates maladaptive repair of renal tubular epithelial cells by restoring CHMP2A-mediated phagosome closure
Source: Cell Death Dis. 2021 Nov 16;12(12):1087. doi: 10.1038/s41419-021-04372-6 (PMC8599682; doi:10.1038/s41419-021-04372-6)
Supplement: Supplementary file 1 — Supplementary material [file 41419_2021_4372_MOESM1_ESM.pdf]

Supplementary information (SI)

Supplementary Figures (SFig.)

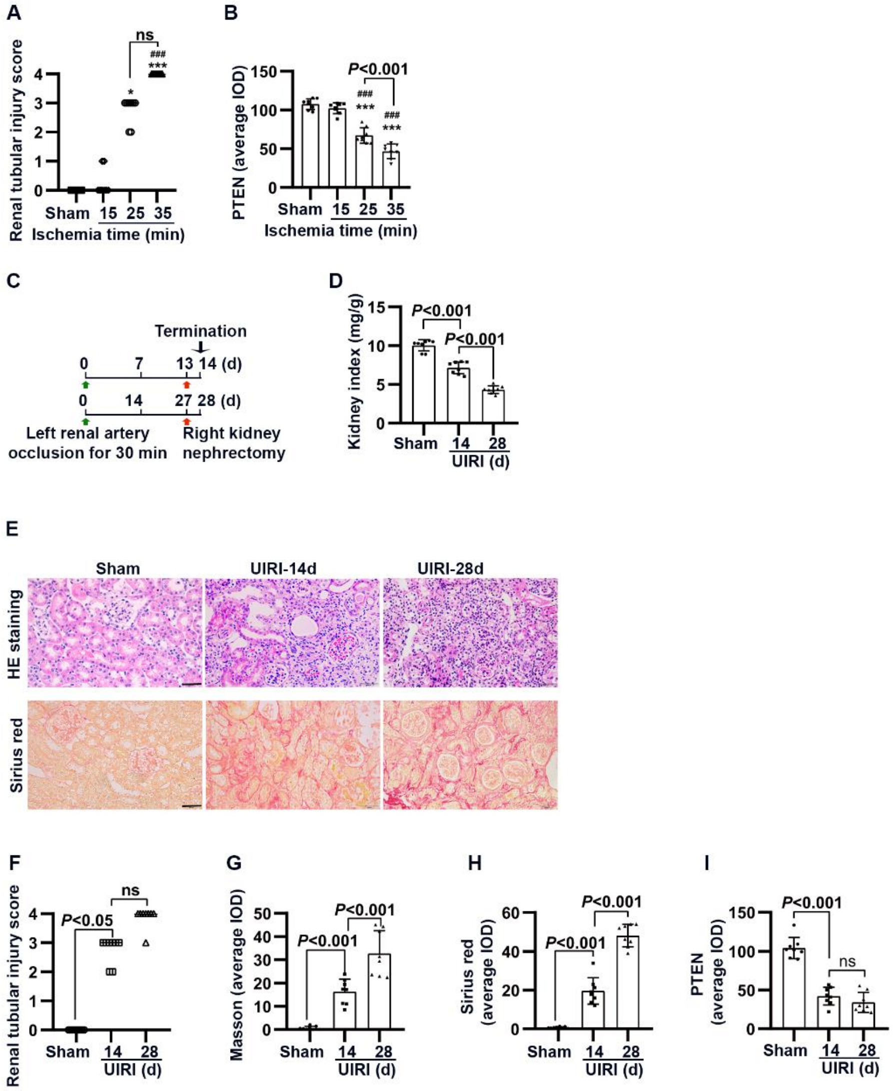

Supplementary Figure 1 (SFig. 1)

(A) Quantification of renal tubular injury in mice subjected to bilateral renal artery obstruction at indicated times, followed by 24 hours reperfusion (BIRI). Renal tubular injury was examined by the percentage of tubular necrosis, cellularcasts, and damaged tubules. Score 0, 1, 2, 3 and 4 represent the injury involving less than 10%, 10-25%, 25-50%, 50-75% and > 75% of the kidney tissue area, respectively. The renal tubular injury scores are presented as a class variable and analyzed using KruskalWallis nonparametric test ($^{*}P$<0.05 and $^{***}P$<0.001 compared with values in sham group; $^{\#\#\#}P$<0.001 compared with values in BIRI-15 min ischemia group; n=8 mice in each group). (B) Quantification of PTEN-positive immunohistochemical staining area in mice subjected to BIRI ($^{***}P$<0.001 compared with values in sham group; $^{\#\#\#}P$<0.001 compared with values in BIRI-15 min ischemia group; n=8 mice in each group). (C) Unilateral renal artery obstruction combined with contralateral uninephrectomy was utilized for renal maladaptive repair model (UIRI). (D) Quantification of the ratio of kidney weight to body weight, termed the kidney index, in mice subjected UIRI. (E) Representative HE and Sirius red staining in kidney subjected to UIRI (scale bar, 50 μm). (F)

Quantification of renal tubular injury in mice subjected to UIRI. (G) Quantification of Masson-positive areas in mice subjected to UIRI. (H) Quantification of Sirius red-positive areas in mice subjected to UIRI. (I) Quantification of PTEN-positive immunohistochemical staining areas in mice subjected to UIRI (n=8 mice in each group).

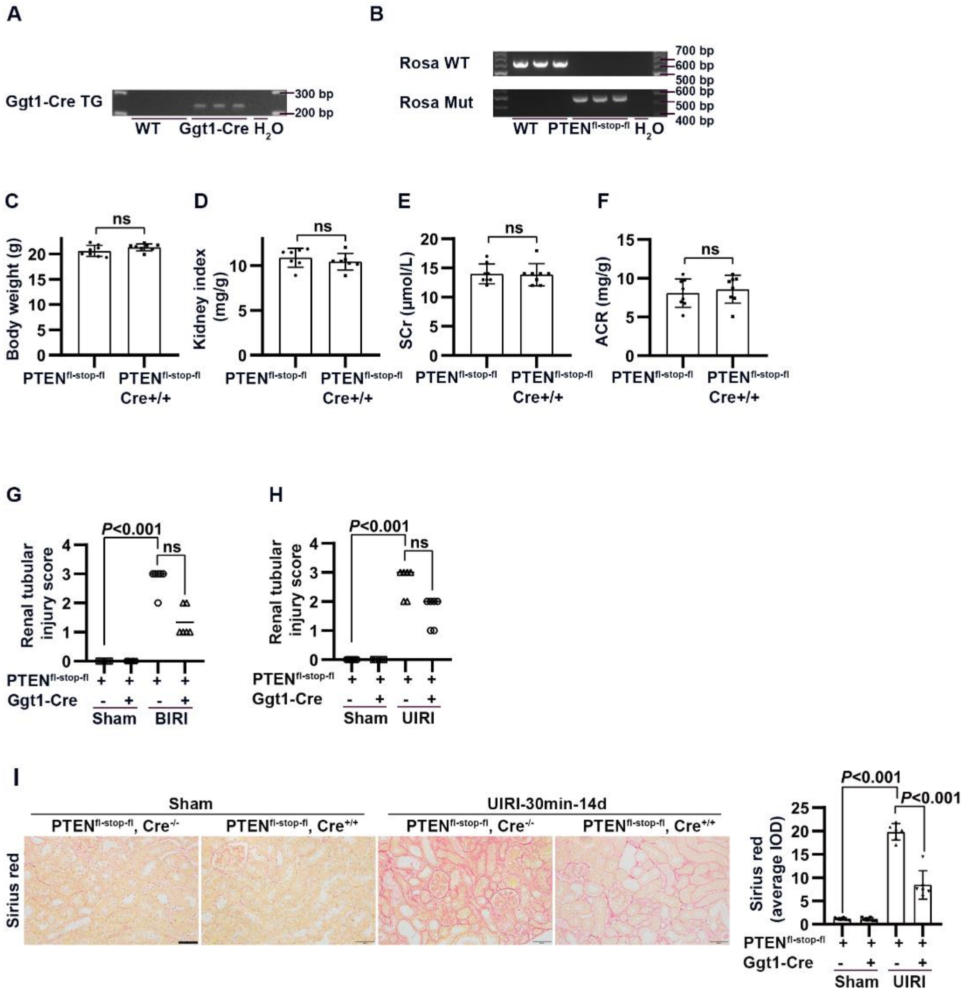

Supplementary Figure 2 (SFig. 2)

(A) Identification of the genotype Ggt1-Cre by Northern blotting. (B) Identification of the genotype PTEN$^{fl\text{-}stop\text{-}fl}$ by Northern blotting. (C-F) Body weight, kidney index (calculated as the ratio of kidney weight to body weight), serum creatinine, and urinary albumin creatinine ratio (ACR) of PTEN$^{fl\text{-}stop\text{-}fl}$, Cre$^{-/-}$ and PTEN$^{fl\text{-}stop\text{-}fl}$, Cre$^{+/+}$ mice. (G) Quantification of renal tubular injury in PTEN$^{fl\text{-}stop\text{-}fl}$, Cre$^{-/-}$ and PTEN$^{fl\text{-}stop\text{-}fl}$, Cre$^{+/+}$ mice subjected to BIRI. (H) Quantification of renal tubular injury in PTEN$^{fl\text{-}stop\text{-}fl}$, Cre$^{-/-}$ and PTEN$^{fl\text{-}stop\text{-}fl}$, Cre$^{+/+}$ mice subjected to UIRI. (I) Representative images and quantification of Sirius red staining in kidney in PTEN$^{fl\text{-}stop\text{-}fl}$, Cre$^{-/-}$ and PTEN$^{fl\text{-}stop\text{-}fl}$, Cre$^{+/+}$ mice subjected to UIRI (scale bar, 50 μm).

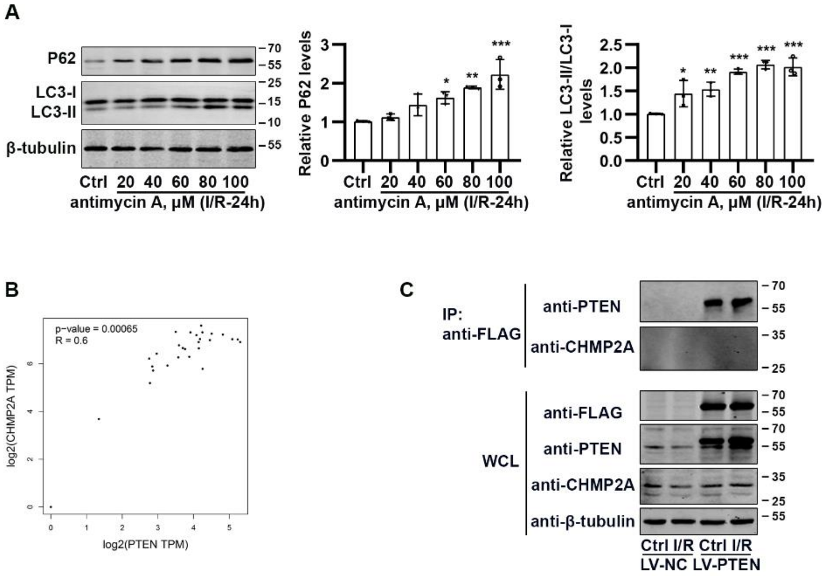

Supplementary Figure 3 (SFig. 3)

(A) Western blotting analysis showing changes in LC3 and P62 in I/R-treated HK-2 cells under an antimycin A concentration gradient ($^{*}P$<0.05, $^{**}P$<0.01, and $^{***}P$<0.001 compared with values in the Ctrl group). (B) Correlation analysis of PTEN and CHMP2A in Kidney-Cortex from Genotype-Tissue Expression database (GTEx). From: http://gepia2.cancer-pku.cn/#correlation. (C) Co-immunoprecipitation assay was performed in I/R-treated HK-2 cells in the absence and presence of LV-PTEN that using a FLAG antibody as the bait, and analyzing by western blotting using PTEN and CHMP2A antibody.
